# Tissue Transglutaminase in Marmoset Experimental Multiple Sclerosis: Discrepancy between White and Grey Matter

**DOI:** 10.1371/journal.pone.0100574

**Published:** 2014-06-24

**Authors:** Nathaly Espitia Pinzon, Esther Stroo, Bert A. ‘t Hart, John G. J. M. Bol, Benjamin Drukarch, Jan Bauer, Anne-Marie van Dam

**Affiliations:** 1 VU University Medical Center, Neuroscience Campus Amsterdam, Department of Anatomy and Neurosciences, Amsterdam, The Netherlands; 2 Biomedical Primate Research Center, Department of Immunobiology, Rijswijk, The Netherlands; 3 University Groningen, University Medical Center, Department of Neuroscience, Groningen, The Netherlands; 4 Center for Brain Research, Department of Neuroimmunology, Vienna, Austria; Medical University Vienna, Center for Brain Research, Austria

## Abstract

Infiltration of leukocytes is a major pathological event in white matter lesion formation in the brain of multiple sclerosis (MS) patients. In grey matter lesions, less infiltration of these cells occur, but microglial activation is present. Thus far, the interaction of β-integrins with extracellular matrix proteins, e.g. fibronectin, is considered to be of importance for the influx of immune cells. Recent in vitro studies indicate a possible role for the enzyme tissue Transglutaminase (TG2) in mediating cell adhesion and migration. In the present study we questioned whether TG2 is present in white and grey matter lesions observed in the marmoset model for MS. To this end, immunohistochemical studies were performed. We observed that TG2, expressed by infiltrating monocytes in white matter lesions co-expressed β_1_-integrin and is located in close apposition to deposited fibronectin. These data suggest an important role for TG2 in the adhesion and migration of infiltrating monocytes during white matter lesion formation. Moreover, in grey matter lesions, TG2 is mainly present in microglial cells together with some β_1_-integrin, whereas fibronectin is absent in these lesions. These data imply an alternative role for microglial-derived TG2 in grey matter lesions, e.g. cell proliferation. Further research should clarify the functional role of TG2 in monocytes or microglial cells in MS lesion formation.

## Introduction

Multiple Sclerosis (MS) is a chronic, inflammatory demyelinating disease of the human central nervous system (CNS), affecting mostly young adults in the prime of their lives [1]. Its clinical manifestation is characterized mainly by motor and sensory deficits, and most commonly has a relapsing-remitting course [2], [3]. Although there is a debate on the immunological versus neurodegenerative origin of MS [4]–[6], it is well-established that the entry of leukocytes into the CNS is an important event in the pathophysiology of MS [7], [8], in addition to glial cell activation [9]–[12]. In active white matter MS lesions, a disturbance of the blood-brain barrier function permits this influx of immunomodulatory cells, contributing to inflammation, demyelination and axonal damage evoking neurological deficits [13], [14]. In grey matter lesions, the influx of immunomodulatory cells is rather limited, whereas activated microglial cells are present like in white matter lesions but to a lesser extent [15]–[22].

During MS white matter lesion formation, basement membranes, i.e. thin layers of connective tissue lining the perivascular space, and the brain parenchyma express various types of extracellular matrix (ECM) protein deposits, such as fibronectin (FN), an important ECM protein in MS lesions [23]–[25]. ECM proteins are generally important because they play a role in the recruitment of inflammatory cells by interacting with integrins expressed on activated leukocytes [26]–[28]. This interaction occurs via the recognition site amino acid motif Arg-Gly-Asp (RGD) that can be found within FN [29] and many other matrix proteins [30]. Of the integrins, α_5_β_1_-integrin is the major cell surface integrin interacting with the RGD-cell binding site on FN, facilitating cell adhesion [31]. Of additional interest in this process is the multifunctional Ca^2+^-dependent enzyme tissue Transglutaminase (TG2). TG2 is expressed in the cytoplasm or surface of a wide variety of cells, and can be deposited in the ECM [32]. This enzyme, when activated, is able to bind and cross-link several ECM proteins, though its interaction with FN is best characterized [33]. More recently, it has become clear that various β-integrins can interact with TG2, forming β-integrin-TG2 complexes on the cell surface [34]–[36]. Consequently, TG2 is referred to as an integrin-binding coreceptor for FN [37]. In this manner, TG2 can contribute to cell-matrix interactions such as cell adhesion and possibly other β-integrin-dependent functions including cell spreading and migration of e.g. monocytes [38]–[40] that likely are of importance during MS lesion formation. In the present study, we therefore question whether TG2 is present in various lesion types in experimental autoimmune encephalomyelitis (EAE) in the common marmoset. This experimental animal model mimics relevant clinical symptoms and relevant inflammatory, glial, and demyelinating white and grey matter pathology associated with relapsing-remitting MS [5], [41] which is uncommon in rodent models for MS [42], [43]. To this end, we studied the presence of immunoreactive TG2 in white and grey matter lesions of marmosets suffering from EAE, identified the cell types expressing TG2, and related those to FN and β_1_-integrin expression.

## Materials and Methods

### Brain material from marmosets

For this study we obtained, with permission, brain material from marmosets (*Callithrix jacchus*) suffering from EAE, that had been involved in preclinical experiments on the refinement of the experimental autoimmune encephalomyelitis model (see also Table 1) [44]. The original studies were approved by the BPRC committee on Animal Experimentation (DEC; approval numbers 483, 512, 514), and carried out in strict accordance with their guidelines. In that study, all marmosets were housed in pairs in spacious cages enriched with branches and toys, and with padded shelter provided on the floor. They remained under veterinary care and clinical scoring was performed twice daily by trained observers, using a previously described semiquantitative scale [45]. The animals were sacrificed once their clinical signs reached the score of 2.5, e.g. paresis. We did not perform any animal experiments for this present study.

**Table 1:** Lesion types per animal.

Animal	Immunization antigen	Lesion type (number per type)
1	MOG_34–56_	IA (5) + LA (2)
2	MOG_34–56_	EA (2) + IA (1) + LA (2)
3	MOG_34–56_	LA/IA (3) + EA/LA (1)
4	MOG_34–56_	LA (1)
5	MOG_74–96_ + MOG_34–56_	EA/LA (1) + IA (1) + cGML (1)
6	MOG_74–96_ + MOG_34–56_	EA/LA (1) + LA/IA (1) + IA(1)
7	MOG_74–96_ + MOG_34–56_	LA/IA (1) + IA (1)
8	MOG_74–96_ + MOG_34–56_	EA/LA (1) + cGML (2)

MOG: myelin oligodendrocyte glycoprotein, EA: early active, LA: late active, IA: inactive, cGML: cortical grey matter lesion.

### Histopathology

From formalin-fixed paraffin-embedded brains, coronal sections (3–5 µm) were cut and used for immunohistochemistry. Brain sections were deparaffinized by heating them at 56°C for 30 min. Sections were then rinsed three times for 10 min. in clear advantage (xylene replacement, Polyscience Inc., Warrington, United States) and subsequently immersed for 5 min. each in 100% ethanol (twice), 96% ethanol, 90% ethanol, 70% ethanol and demineralized water. The extent of inflammation was evaluated by staining for hematoxylin and eosin to visualize infiltrated cells and a staining for myeloid-related protein 14 (MRP14, BMA Biomedicals, Augst, Switzerland) was performed to visualize macrophages [46], [47]. Moreover, a Klüver Barrera stain (Luxol Fast Blue (LFB) combined with periodic acid-Schiff (PAS)) was performed to examine myelin and myelin degradation products as previously described [45]. Images were taken using an Olympus-VANOX-T lightmicroscope (Tokyo, Japan).

### TG2 immunoreactivity

After deparaffination, antigen retrieval was performed by incubating the sections in ethylenediaminetetraacetic acid (EDTA, pH = 9.0) buffer for 30 min. in a steaming device (MultiGourmet FS 20; Braun, Kronberg/Taunus,Germany). Subsequently, the sections were allowed to regain room temperature (RT), washed three times in Tris-buffered saline (TBS, pH 7.4), 5 min. each, and endogenous peroxidase was blocked for 20 min. with 0.3% hydrogen peroxidase and 0.1% sodium azide in TBS. Sections were washed three times again with TBS for 5 min each. Non-specific binding sites were blocked with DAKO buffer (0.05 M Tris/HCl, 0.15 M NaCl, 0.05% Tween 20, pH 7.6, DAKO, Glostrup, Denmark) with 10% fetal calf serum (FCS) for 30 min. at room temperature. For TG2 staining, the sections were subsequently incubated overnight at 4°C with mouse anti TG2 (Ab3, Neomarkers; final dilution 1∶15,000) diluted in DAKO buffer with 10% FCS. After washes in TBS, the sections were incubated for 2 hrs at RT in biotinylated donkey anti-mouse IgG (Jackson Laboratories; final dilution 1∶500). After washes in TBS, the sections were incubated for one hour in HRP-labeled avidin-biotin complex (1∶100; Sigma, St. Louis, USA). Sections were washed twice with TBS and once with Tris-HCl (pH 7.6). Peroxidase activity was visualized by adding 3,3-diaminobenzidine (DAB, Sigma) as a substrate. Sections were washed twice with Tris-HCl and once with running tap water. Finally, sections were counterstained with haematoxylin and sections were washed three times in running tap water. After dehydration in graded ethanol solutions, the sections were cleared in xylene and coverslipped in Entellan (Merck, Darmstadt, Germany). Images were taken using an Olympus-VANOX-T light microscope.

### Fluorescent double labeling procedures

Three different protocols were used for double labeling of TG2 with various antigens, decided by the fluorescent intensity of the stainings. After the preincubation step as described above, the sections for (**1**) double labeling of FN or the astrocyte marker glial fibrillary acidic protein (GFAP) with TG2 were incubated with the appropriate primary antibodies in 5% normal donkey serum in 0.5% Triton-X100 (TBS-T; pH 7.6, blocking solution) at 4°C overnight (see Tables 2 and 3). Subsequently, the sections were thoroughly washed in TBS, and incubated at room temperature for 2 hrs with appropriate Alexa Fluor 488 or Alexa Fluor 546, and Alexa Fluor 594 labeled IgG's (see Tables 2 and 4); (**2**) double labeling of the B-cell marker CD20 or the oligodendrocytes marker Olig2 with TG2 were incubated with the appropriate primary antibodies in 5% normal donkey serum in TBS-T at 4°C overnight (see Tables 2 and 3). Thereafter, the sections were thoroughly washed in TBS, and incubated at room temperature for 2 hrs with appropriate biotinylated labeled IgG's for CD20 or Olig2 (see Tables 2 and 4) and concomitantly with the appropriate Alexa Fluor 594 labeled IgG to stain for TG2. Thereafter, the sections were washed in TBS and finally incubated for 2 hrs at room temperature with Alexa Fluor 488 labeled-streptavidin (1∶400, Molecular Probes, Breda, the Netherlands) to detect CD20 or Olig2; (**3**) double labeling of β_1_-integrin, the monocyte/microglia Iba-1 or the T-cell marker CD3 with TG2 were incubated with the appropriate primary antibodies in 5% normal donkey serum in TBS-T at 4°C overnight (see Tables 2 and 3). Sections were then thoroughly washed, and incubated at room temperature for 2 hrs with the appropriate biotinylated labeled IgG's for β_1_-integrin, Iba-1 or CD3 (see Tables 2 and 4) and concomitantly with the appropriate Alexa Fluor 594 labeled IgG to detect TG2. Sections were washed in TBS and incubated for one hour in ABC (1∶800, ABC kit, Vectastain elite, Vector Laboratories Inc., Burlingame, CA, USA). Sections were then washed in TBS again and incubated with biotinylated tyramide (1∶800, gift from dr. I. Huitinga, The Netherlands Institute for Neuroscience (NIN), Amsterdam, The Netherlands) in 0.005% H_2_O_2_ in TBS for 20 min. Sections were washed once more in TBS and incubated once more for 1 hr in ABC (1∶800) and washed in TBS again. The last step in this adjusted protocol was incubation of the sections with Alexa Fluor 488 labeled-streptavidin (1∶400, Molecular Probes) for 2 hrs to detect β_1_-integrin, Iba-1 or CD3. Finally, at the end of all 3 double labeling protocols, sections were washed in TBS and mounted in Vectashield (Vector laboratories Inc.). Immunofluorescence was examined using a Leica confocal laser scanning microscope (Leica TSC-SP2-AOBS; Leica Microsystems, Wetzlar, Germany). Omission of the primary antibodies served as a negative control.

**Table 2:** Combinations of primary and secondary antibodies used for immunoreactive labeling.

Primary antibodies (see table 3)	Host	Dilutions	Secondary antibodies (see table 4)
Ab3 + β_1_-integrin	Mouse + Rabbit	1∶1000+1∶1000	2+6
Ab3 + CD3	Mouse + Rabbit	1∶1000+1∶800	2+6
Ab3 + FN	Mouse + Sheep	1∶1000+1∶100	2+7
Ab3 + GFAP	Mouse + Rabbit	1∶1000+1∶2000	2+4
Ab3 + Iba-1	Mouse + Goat	1∶1000+1∶600	2+1
Ab3 + Olig2	Mouse + Rabbit	1∶1000+1∶750	2+6
Ab4 + CD20	Rabbit + Mouse	1∶400+1∶50	5+3

FN: fibronectin.

**Table 3:** Origin of primary antibodies used.

Antigen	Host	Manufacturer
Transglutaminase type 2 (Ab3)	Mouse	NeoMarkers
Transglutaminase type 2 (Ab4)	Rabbit	NeoMarkers
β_1_-integrin	Rabbit	Santa Cruz
CD3 (pan T-lymphocytes)	Rabbit	DAKO
CD20 (pan B-lymphocytes)	Mouse	DAKO
Fibronectin	Sheep	R&D systems
GFAP (astrocytes)	Rabbit	DAKO
Iba-1 (monocytes/macrophages/microglia)	Goat	Abcam
Olig2 (oligodendrocytes)	Rabbit	Millipore

**Table 4:** Secondary antibodies used.

Number	Host	Target	Dilution	Labeled	Manufacturer
1	Donkey	Goat	1∶800	Biotin	Jackson
2	Donkey	Mouse	1∶400	Alexa-594	Mol. Probes
3	Goat	Mouse	1∶800	Biotin	Jackson
4	Donkey	Rabbit	1∶400	Alexa-488	Mol. Probes
5	Donkey	Rabbit	1∶400	Alexa-594	Mol. Probes
6	Goat	Rabbit	1∶800	Biotin	Jackson
7	Donkey	Sheep	1∶400	Alexa-546	Mol. Probes

### Quantification of TG2 and Iba-1 positive cells

The number of TG2 and Iba-1 positive cells was quantified in inactive white matter lesions (12 lesions, from 7 different animals) and in early/late active lesions (8 lesions, from 6 different animals). Of each lesion, one representative image per 40X microscopic field was taken with a Leica confocal laser scanning microscope. Images were of equal sized (0.1 mm^2^) random sample areas. Cells in these images were counted using Cell∧F Olympus Soft Imaging Solutions GmbH software (Tokyo, Japan).

### Statistics

Data were analyzed by a Student's t-test for unpaired independent measurements by using the SPSS 15.0 for Windows statistical program (SPSS, Inc., Chicago IL). P<0.05 was considered to represent statistically significant differences.

## Results

### Characterization of marmoset EAE lesions

**Figure 1:**
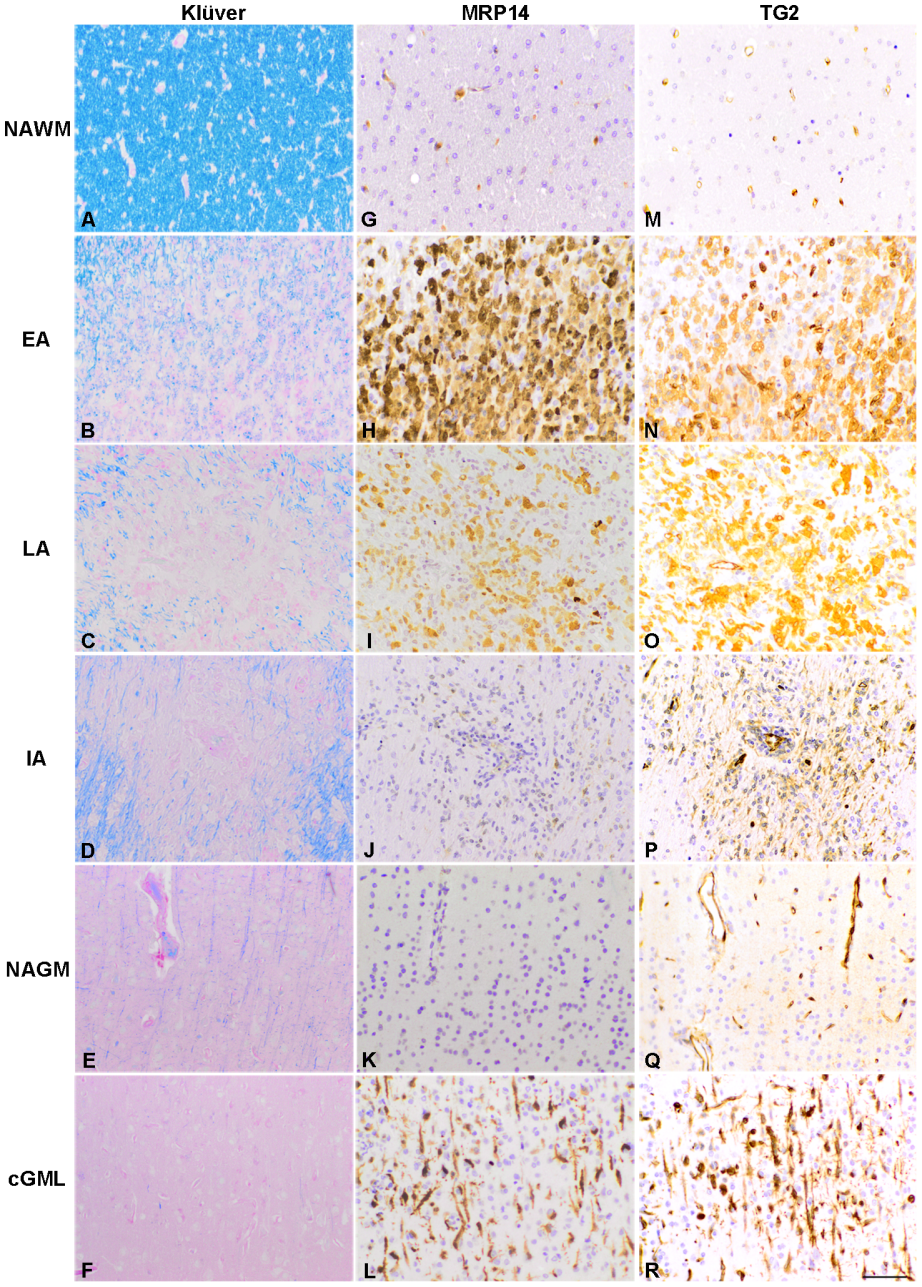
Characterization of marmoset EAE lesions and TG2 immunoreactivity. The normal appearing white matter (NAWM) shows an intact LFB myelin staining (A) and few MRP14 positive macrophages (G). Early active (EA) lesions display myelin degradation (B) and foamy macrophages (H). Late active (LA) lesions show degradation of myelin (C) combined with less MRP14 positive macrophages (I). Inactive (IA) lesions are characterized by an absence of both myelin staining (D) and MRP14 positive macrophages (J). The normal appearing grey matter (NAGM) shows intact myelin fibers (E) and very few MRP14 positive macrophages (K). Cortical grey matter lesions (cGML) show an absence of myelin fibers (F) and presence of MRP14 positive microglia (L). TG2 immunoreactivity is present in endothelium of the vessel walls in NAWM (M). Early active and late active lesions display additional TG2 positive cells (N and O respectively). Inactive lesions show less additional TG2 immunoreactivity (P). Cortical grey matter lesions also show additional TG2 positive cells (R) compared to the endothelial staining in normal appearing grey matter (Q). Scale bar is 20 µm.

Normal appearing white and grey matter were defined by an intact myelin staining and few MRP14 positive macrophages (Fig. 1A, G and Fig. 1E, K respectively) [46]. Early active white matter lesions were characterized by the abundant presence of macrophages with LFB positive myelin degradation products (Fig. 1B, H). Late active white matter lesions were identified by the presence of macrophages containing PAS positive myelin degradation and residual LFB positive myelin degradation products together with a diminished presence of MRP14 positive macrophages (Fig. 1C, I). Inactive white matter lesions were identified by the presence of some PAS positive macrophages in the absence of LFB and MRP14 positive macrophages (Fig. 1D, J). Cortical grey matter lesions were characterized by in the absence of myelin staining and an increase in MRP14 positive microglial cells (Fig. 1F, L). The number and type of EAE lesions varied between and within animals (Table 1).

### TG2 immunoreactivity is present in marmoset EAE lesions

**Figure 2:**
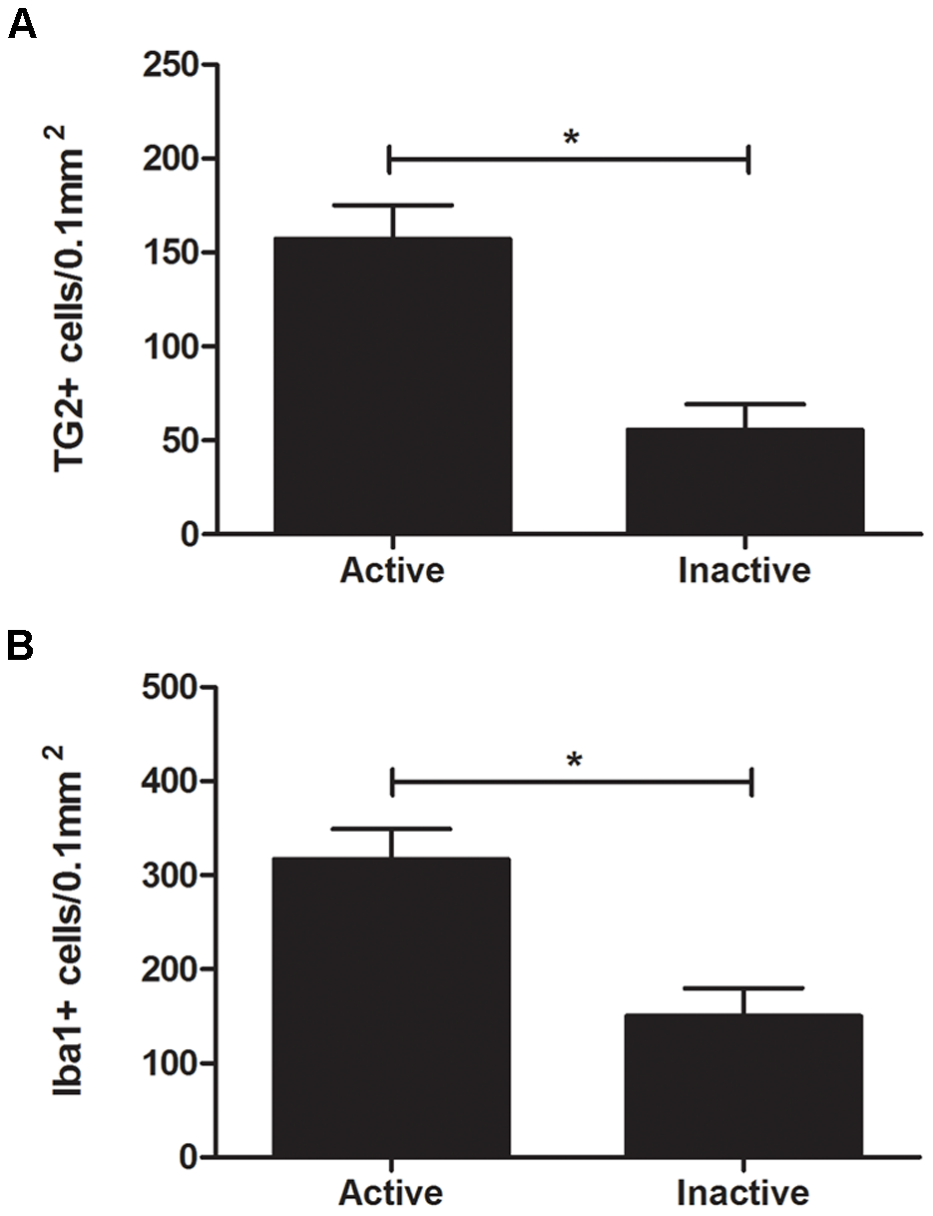
Quantification of TG2 and Iba-1 positive cells in early/late active versus inactive white matter lesions. The number of TG2 (A) and Iba-1 positive cells (B) per white matter sample area of 0.1 mm^2^ is significantly decreased in inactive lesions compared to (early/late) active lesions. Data are shown as mean + SEM, n = 8 for early/late active lesions, n = 12 for inactive lesions, *P<0.001.

In normal appearing white and grey matter, TG2 immunoreactivity was hardly present except in the endothelium of the vessel walls (Fig. 1M, Q), as described before in rodent and human brain [48]–[51]. In the various EAE white matter lesions types, additional TG2 immunoreactivity was observed in cells with a rounded morphology located mostly near blood vessels. The signal intensity of TG2 immunoreactivity was not uniform in all positive cells, suggesting different TG2 expression levels. Early and late active lesions showed a considerable amount of TG2 immunoreactive cells throughout the lesion (Fig. 1N, O). In inactive lesions less TG2 positive cells appeared to be present (Fig. 1P). Indeed, after quantification, the number of TG2 positive cells per white matter sample area of 0.1 mm^2^ in early/late active lesions was about 3 times higher than in inactive lesions (Fig. 2A). In addition, TG2 immunoreactivity appeared in cortical grey matter lesions (Fig. 1R). The majority of TG2 positive cells showed a small cell body with thin, radially projecting processes.

### TG2 is expressed by monocytes/microglial cells in marmoset EAE lesions

**Figure 3:**
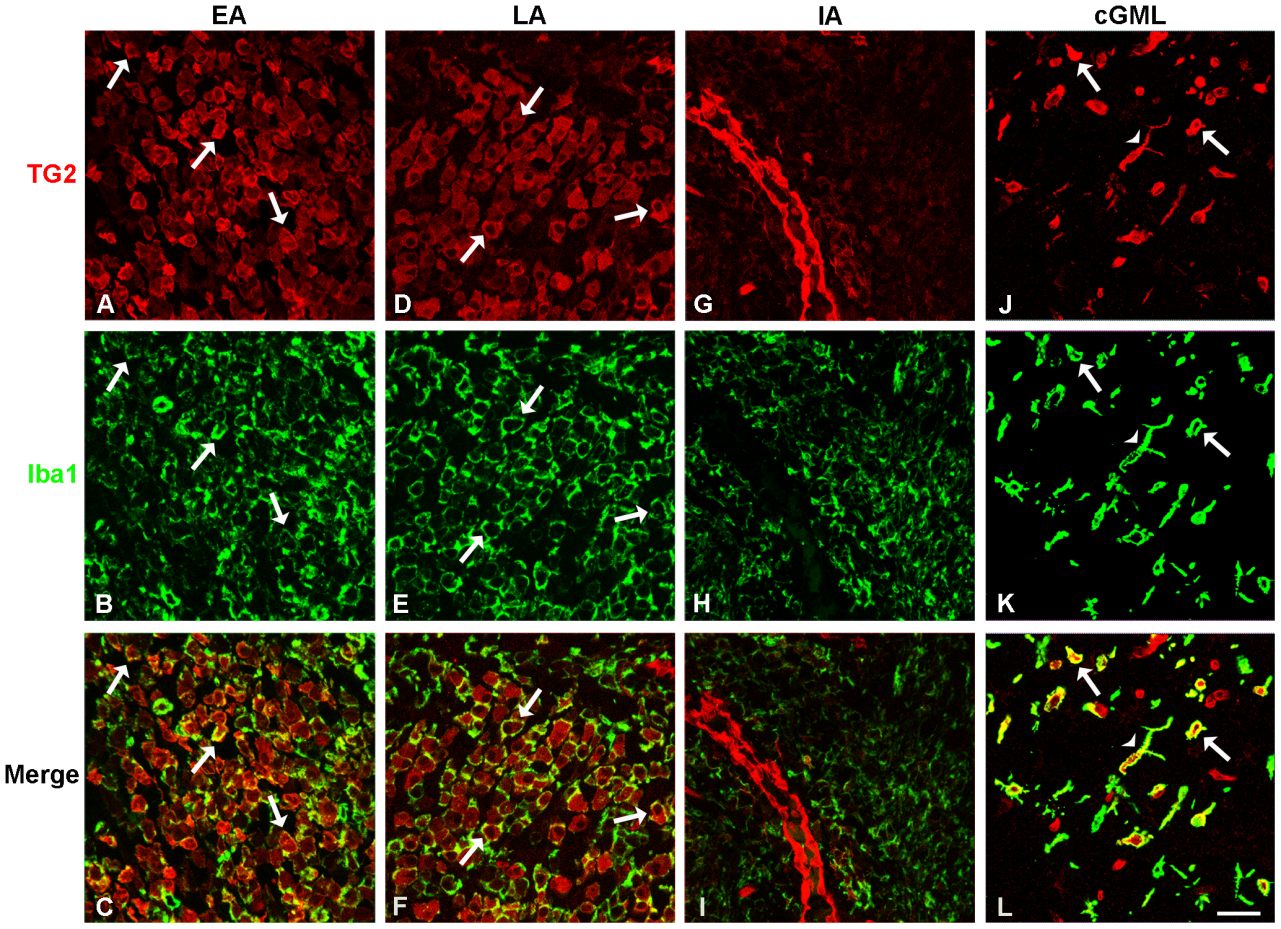
TG2 positive cells show co-labeling with Iba-1 in marmoset EAE lesions. Early (EA) (A-C) and late active (LA) (D-F) lesions show mostly cytoplasmic cellular localization of immunoreactive TG2 (A, D), and cells with membrane labeled Iba-1 (B, E). TG2 positive cells co-label with Iba-1 positive cells (C, F). Inactive (IA) (G-I) lesions show less TG2 positive cells and co-labeling with Iba-1 positive cells seems less apparent (I) Cortical grey matter lesions (cGML) (J-L) show TG2 positive cells co-labeling with Iba-1 positive cells that have radially projecting processes (L), instead of the more rounded morphology seen in white matter lesions (C, F). Arrows represent monocyte-like cells that are either single (top 2 rows) or double labeled (merge), arrowheads represent microglial cells that are either single (top 2 rows) or double labeled (merge). Scale bar is 20 µm.

Iba-1 positive cells were observed in all types of EAE lesions (Fig. 3B, E, H, K). These cells showed Iba-1 immunoreactivity on the cell surface as described previously [52], [53]. TG2 immunoreactivity was mainly localized in the cytoplasm (Fig. 3A, D, G, J). Co-labeling of Iba-1 with TG2 showed Iba-1 positive/TG2 positive cells with a rounded morphology with no clear processes in early (Fig. 3C) and late active white matter lesions (Fig. 3F). Note that not all Iba-1 positive cells express TG2. Based on the morphology of the TG2 positive/Iba-1 positive cells we cannot determine whether these are microglial cells with an amoeboid morphology or infiltrating monocytes, although their localization close to a blood vessel favors the latter option. After quantification, we determined that the number of Iba-1 positive cells per white matter sample area of 0.1 mm^2^ in early/late active lesions was about 2 times higher than in inactive lesions (Fig. 2B). Additionally, co-labeling of Iba-1 with TG2 seemed less apparent in inactive EAE lesions (Fig. 3I) compared to the co-labeling observed in the active EAE lesions, which is likely a consequence of the reduced number of TG2 and Iba-1 positive cells (Fig 2). Co-labeling of TG2 with Iba-1 positive cells was also found in cortical grey matter lesions (Fig. 3L). Interestingly, besides the presence of some TG2 positive monocyte-like cells, the morphology of the majority of TG2/Iba-1 positive cells was largely different from those seen in the white matter lesions, and reflected more ramified microglial cells (Fig. 1R).

### TG2 is not expressed in astrocytes, oligodendrocytes, T- and B-lymphocytes

**Figure 4:**
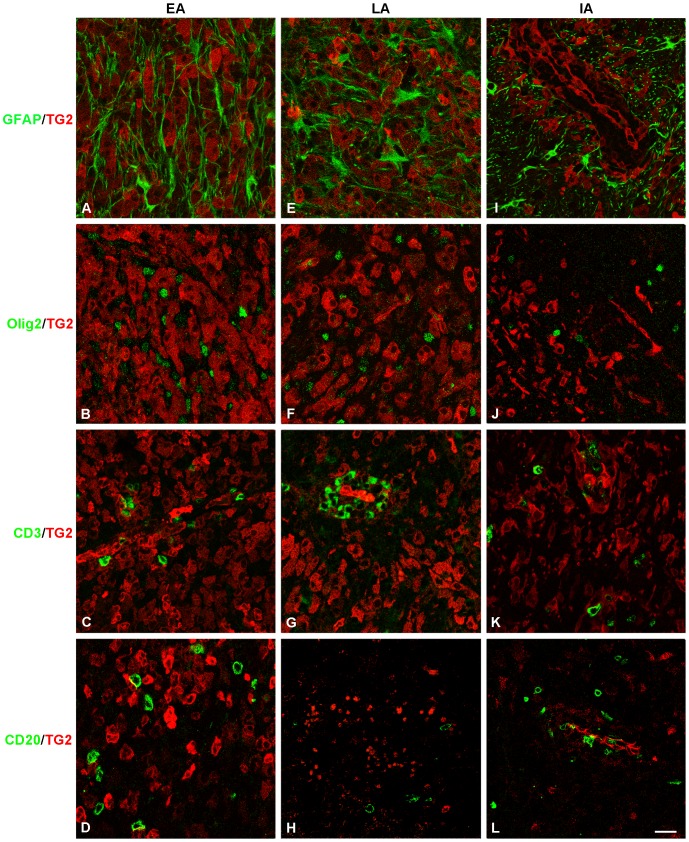
TG2 is not expressed in astrocytes, oligodendrocytes, T-cells and B-cells. TG2 immunoreactivity (red) is not present in GFAP (green; A, E, I), Olig2 (green; B, F, J), CD3 (green; C, G, K) or CD20 (green; D, H, L) positive cells in early (EA), late active (LA) and inactive (IA) lesions. Scale bar is 20 µm.

To examine whether also non-myeloid cell types expressed TG2 in various marmoset EAE white matter lesions, additional immunofluorescent double labeling experiments were performed. Co-labeling for TG2 and GFAP was absent, indicating that astrocytes did not express TG2 in EAE lesions (Fig. 4A, E, I). Similarly, Olig2 positive oligodendrocytes appeared negative for TG2 (Fig. 4B, F, J). Moreover, TG2 immunoreactivity was neither present in CD3 positive T-lymphocytes (Fig. 4C, G, K) nor in CD20 positive B-lymphocytes (Fig. 4D, H, L).

### β_1_-integrin and FN immunoreactivity in relation to TG2 positive cells in marmoset EAE lesions

**Figure 5:**
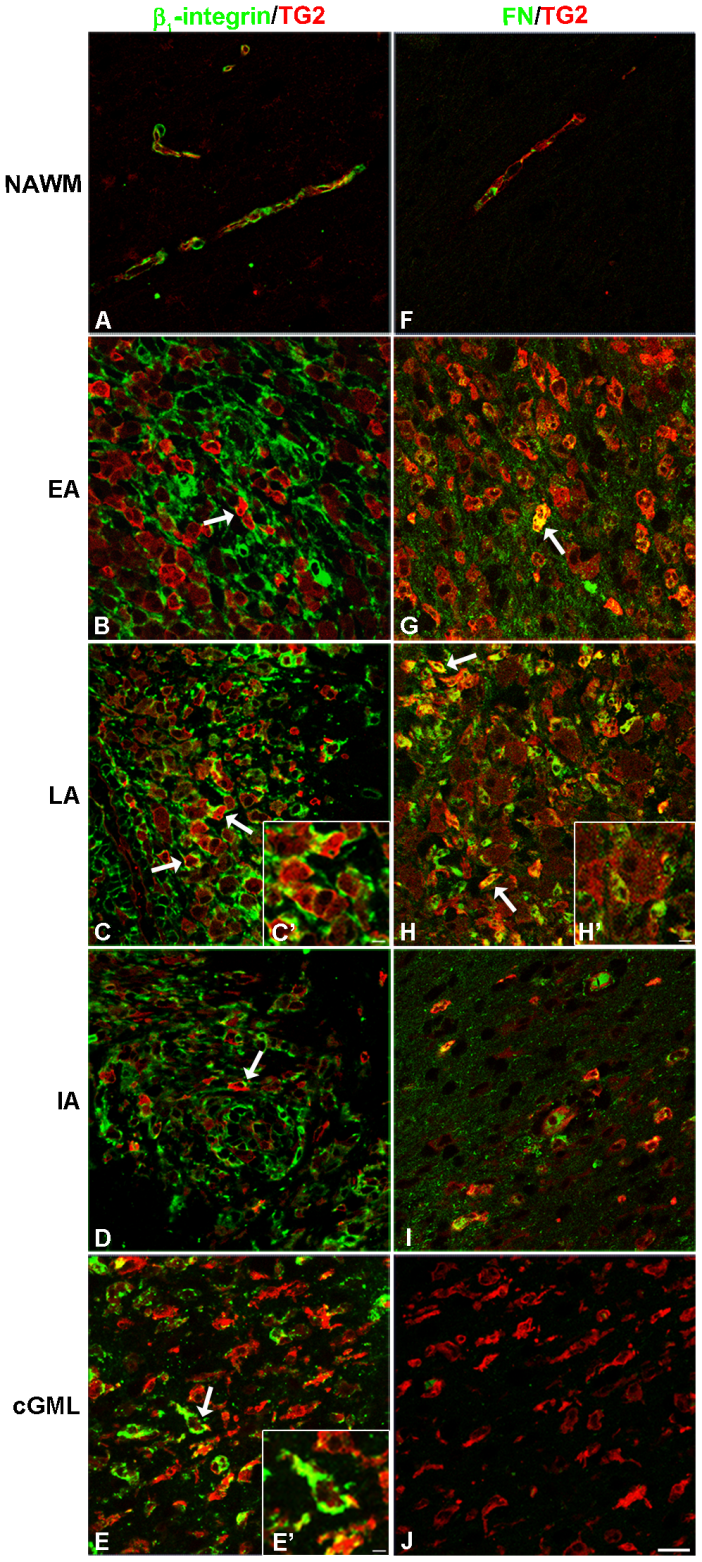
β_1_-integrin and fibronectin immunoreactivity show co-presence with TG2 positive cells in marmoset EAE lesions. β_1_-integrin (A) and fibronectin (FN) (F) immunoreactivity (green) is found in normal appearing white matter (NAWM) near TG2 (red) in the endothelium of the vessel walls. β_1_-integrin (green; B, C, C′, D) appears in early (EA), late (LA) active lesions and inactive (IA) white matter lesions on the cell surface of a subset of TG2 (red) positive cells. β_1_-integrin also shows some co-localization with TG2 positive cells in cortical grey matter lesions (cGML) (E, E′). Arrows represent TG2/β_1_-integrin double labeled cells. FN (green; G, H, H′, I) appears clearly in the extracellular matrix but also shows co-labeling with a subset of TG2 (red) positive cells in early and late active lesions. Hardly any FN immunoreactivity is present in grey matter lesions (J). Arrows represent TG2/FN double labeled cells. Scale bar is 20 µm. Inserts in figures C′, E′ and H′ represent higher magnifications in which the close association of TG2 positive cells with β_1_-integrin or FN (C′, E′ or H′, respectively) can be appreciated. Scale bars in the inserts are 10 µm.

We further studied whether β_1_-integrin and/or FN showed co-labeling with TG2 in marmoset EAE lesions, since TG2 has been referred to as an integrin-binding coreceptor for FN in previous *in vitro* studies [37], [54]. Compared to control (Fig 5A) β_1_-integrin immunoreactivity was increased in all EAE lesion types, (Fig. 5B-D). Double labeling experiments showed the presence of β_1_-integrin particularly on the cell surface of a subset of TG2 positive cells, specifically in active white matter lesions (Fig. 5C, C′). Also in grey matter lesions, β_1_-integrin was present in or on TG2 positive cells. Although not quantified, it appeared that amoeboid-shaped cells expressed more β_1_-integrin than ramified microglial cells (Fig. 5E).

FN immunoreactivity was hardly present in NAWM (Fig. 5F), but was clearly increased in active white matter lesions and increased to a lesser extent in inactive lesions (Fig. 5G-I). FN was found to be partly cell-associated, but was mostly present in the ECM in these lesions (Fig. 5G-I). Double labeling experiments for FN and TG2 showed little co-labeling, but clearly close association of TG2 positive monocytes with extracellular FN in the matrix was seen most prominent in active white matter lesions (Fig. 5H, H′). In grey matter lesions, there was little FN immunoreactivity present (Fig. 5J).

## Discussion

The present study shows appearance of TG2 immunoreactivity in monocyte and microglial-like cells in early active white matter, and active grey matter marmoset EAE lesions. When white matter lesions progress to late active and inactive stages, TG2 immunoreactivity is still present, but in the inactive lesions it is significantly less pronounced. In addition, in white matter lesions, TG2 positive monocytes co-label with β_1_-integrin, and are in close apposition to, mostly extracellular located, fibronectin. In grey matter lesions, TG2 positive microglia co-label with β_1_-integrin, but no fibronectin is present.

For this study we were able to obtain material from marmosets suffering from EAE. This primate has high genetic similarity to humans. Its mature immune system, shaped by life-long exposure to environmental and latent infections, resembles the human immune system. The MS-like disease phenotype and pathology of marmoset EAE is therefore a useful model to investigate if certain factors, in this case TG2, contribute to the pathogenesis of MS [55].

In active white matter lesions, we observed the appearance of the enzyme TG2, particularly located around blood vessels where leukocytes infiltrate into the CNS during the EAE disease process. The number of these TG2 positive cells reduces when the lesions lose activity. This reduction occurs simultaneously with the reduction of Iba-1 positive cells in the CNS at this time [56]–[59]. Based on co-labeling studies, TG2 was found to be present in Iba-1 positive infiltrating monocytes in white matter lesions in marmoset EAE. Moreover, we recently observed major histocompatibility complex (MHC) II positive monocyte-like cells to express TG2 immunoreactivity in active white matter MS lesions (unpubl. data). Thus infiltrating monocytes seem to represent an important source of TG2 in MS/EAE active white matter lesions within the CNS. In grey matter lesions, TG2 appeared preferentially in microglial cells. Microglial-derived TG2 has also been described in gerbil hippocampal grey matter after transient ischemia [60]. This suggests that lesioned grey matter areas express TG2 preferentially in microglial cells. Thus far, TG2 has been shown to be expressed by a wide variety of cell types, both in vivo and in vitro [32]. In the presented marmoset EAE model, the expression of TG2 was selectively found in myeloid cell types, as other cell markers did not co-localize with TG2, excluding the presence of TG2 in astrocytes, oligodendrocytes, T-cells or B-cells. In contrast, in chronic active MS lesions, TG2 has been found in astrocytes [61], which might reflect a pathological difference between marmoset and human disease. Within the human CNS, TG2 was shown to be mainly expressed by neurons under physiological [62] and pathological conditions [63]–[65]. TG2 is considered to play a pathophysiological role in aggregation of pathological/misfolded proteins, including huntingtin, α-synuclein and β-amyloid [66]–[68]. In more recent years, a role for TG2 in inflammatory processes has been explored. Deletion of the TG2 gene in vivo resulted in an altered immune status of mice [69] probably due to altered cytokine regulation in macrophages [70]. Furthermore, septic shock-mediated influx of neutrophils and cytokine production [71], and T-cell mediated EAE was reduced [72] when the TG2 gene was ablated. In vitro studies have elucidated the expression of TG2 in myeloid cells, including macrophages, microglia and dendritic cells [38], [73]–[75]. Our study is the first to demonstrate lesion-dependent expression of TG2 in monocytes and microglial cells during marmoset EAE. We subsequently questioned whether monocyte-derived TG2 could contribute to the adhesion and migration process of the infiltrating monocytes. We observed that β_1_-integrin, involved in cell-cell or cell-matrix interactions, co-localizes with TG2 in or on monocytes. This is in line with the observation that β_1_-integrin plays an important role in the influx of leukocytes, including monocytes, in the marmoset EAE model [76]. Thus, TG2 together with β_1_-integrin could mediate, at least part of, the influx of the TG2 positive monocytes into the CNS during EAE white matter lesion formation. To do so, β_1_-integrin has to interact with its ligand FN via the RGD binding motif [77]. Alternatively, direct interaction of TG2 with FN can occur because TG2 has a high affinity for FN [49]. In our study, we observed co-labeling of TG2 expressing monocytes with β_1_-integrin and we found TG2 positive cells to be in close association with extracellular FN. These in vivo data indicate that both options of interaction of monocytes with FN are possible, and support the idea that TG2 can act as a β-integrin co-receptor for binding to FN [36], [37] and thereby contribute to the influx of monocytes into the CNS during EAE lesion formation. Indeed, downregulation of cell surface TG2 decreased the adhesion of monocytes onto FN and markedly reduced their migration in vitro [54]. Recently, interaction of monocytes with FN has been shown to determine the differentiation potential of these cells [78]. It is well known that TG2 is highly upregulated when monocytes differentiate into macrophages [79], and thus the observed association of TG2/β_1_-integrin positive monocytes with extracellular FN may also suggest a role in local differentiation into e.g. dendritic cells or microglial cells of importance for regulating the local neuroinflammatory response. In contrast, in the grey matter lesions, FN is hardly present, indicative for less FN production, but could also implicate less damage to the blood-brain-barrier that allows plasma fibronectin to enter the CNS [23]. Although imaging of grey matter lesions remains a challenge, improved MRI and PET obtained imaging data thus far indicate the presence of demyelination and activated microglial cells in grey matter areas, but no clear cell infiltration [80]–[82]. Moreover, post-mortem studies show the presence of activated microglial cells in grey matter lesions, but a relative paucity in the influx of leukocytes [18], [83]–[85]. These data suggest that TG2 expressed by microglial cells in active grey matter lesions is probably not implicated in immune cell infiltration or migration. Of interest is that integrin expression has been demonstrated on microglial cells in MS lesions [86], and subsequent in vitro studies revealed a role for β_1_-integrin in microglial cell proliferation [87]. Indeed, in MS lesions, microglial and monocyte proliferation have been observed [88]. We thus propose that the TG2/β_1_-integrin positive microglial cells in the grey matter lesions are or have been prone to proliferation.

In conclusion, the observed appearance of immunoreactive TG2 in monocytes in active white matter lesions during marmoset EAE, in combination with its co-expression with β_1_-integrin and close association to extracellular FN, strongly suggests an important role for TG2 in the adhesion, migration and/or differentiation of infiltrating monocytes during EAE, and possibly MS. The appearance of TG2 in microglial cells in grey matter lesions together with β_1_-integrin, suggests an alternative role, e.g. microglial proliferation. Our novel observations on TG2 expression in white and grey matter lesions in a highly relevant animal model for MS are of interest in better understanding the possible functional implications TG2 may have in the pathogenesis of white and grey matter lesions in MS.
